# Antibody responses within two leading *Plasmodium vivax* vaccine candidate antigens in three geographically diverse malaria-endemic regions of India

**DOI:** 10.1186/s12936-019-3066-6

**Published:** 2019-12-16

**Authors:** Sonal Kale, Chander P. Yadav, Pavitra N. Rao, Sneh Shalini, Alex Eapen, Harish C. Srivasatava, Surya K. Sharma, Veena Pande, Jane M. Carlton, Om P. Singh, Prashant K. Mallick

**Affiliations:** 1 ICMR-National Institute of Malaria Research, Sector 8, Dwarka, New Delhi India; 2 Department of Biotechnology, Kumaun University, Nainital, Uttarakhand India; 3 Department of Biology, Center for Genomics and Systems Biology, New York University, New York, USA; 4 National Institute of Malaria Research Field Unit, Indian Council of Medical Research, National Institute of Epidemiology Campus, Ayapakkam, Chennai, Tamil Nadu India; 5 National Institute of Malaria Research Field Unit, Civil Hospital, Nadiad, Gujarat India; 6 Jigyansha, International Center of Excellence for Malaria Research, Sector 1, Rourkela, Odisha India

**Keywords:** *Plasmodium vivax*, PvAMA-1, PvMSP-1_19_, Antibody response, Seroprevalence, India

## Abstract

**Background:**

Identifying highly immunogenic blood stage antigens which can work as target for naturally acquired antibodies in different eco-epidemiological settings is an important step for designing malaria vaccine. Blood stage proteins of *Plasmodium vivax*, apical membrane antigen-1 (PvAMA-1) and 19 kDa fragment of merozoite surface protein (PvMSP-1_19_) are such promising vaccine candidate antigens. This study determined the naturally-acquired antibody response to PvAMA-1 and PvMSP-1_19_ antigens in individuals living in three geographically diverse malaria endemic regions of India.

**Methods:**

A total of 234 blood samples were collected from individuals living in three different eco-epidemiological settings, Chennai, Nadiad, and Rourkela of India. Indirect ELISA was performed to measure human IgG antibodies against recombinant PvAMA-1 and PvMSP-1_19_ antigens. The difference in seroprevalence and factors associated with antibody responses at each site was statistically analysed.

**Results:**

The overall seroprevalence was 40.6% for PvAMA-1 and 62.4% for PvMSP-1_19_. Seroprevalence to PvAMA-1 was higher in Chennai (47%) followed by Nadiad (46.7%) and Rourkela (27.6%). For PvMSP-1_19_, seroprevalence was higher in Chennai (80.3%) as compared to Nadiad (53.3%) and Rourkela (57.9%). Seroprevalence for both the antigens were found to be higher in Chennai where *P. vivax* is the dominant malaria species. In addition, heterogeneous antibody response was observed for PvAMA-1 and PvMSP-1_19_ antigens at each of the study sites. Two factors, age and malaria positivity were significantly associated with seropositivity for both the antigens PvAMA-1 and PvMSP-1_19_.

**Conclusion:**

These data suggest that natural acquired antibody response is higher for PvMSP-1_19_ antigen as compared to PvAMA-1 antigen in individuals living in three geographically diverse malaria endemic regions in India. PvMSP-1_19_ appears to be highly immunogenic in Indian population and has great potential as a malaria vaccine candidate. The differences in immune response against vaccine candidate antigens in different endemic settings should be taken into account for development of asexual stage based *P. vivax* malaria vaccine, which in turn can enhance malaria control efforts.

## Background

*Plasmodium vivax* is the most widespread human malaria parasite and a major contributor to the malaria burden outside Africa, accounting for approximately 100 million cases each year [1]. In India, the total number of confirmed malaria cases and death has been decreased in the past, but still it accounts for 52% of deaths outside of the World Health Organization (WHO) African Region [2]. As India has planned to eliminate malaria by 2030 [3], there is a need to strengthen malaria control strategies to achieve this goal. An effective malaria vaccine, which can work in diverse malaria endemic regions and provide protection against the parasite, will greatly decrease the burden of disease.

The blood stage antigens, primary target of natural acquired immunity, responsible for malaria symptoms and pathology are the main target for the malaria vaccine development [4]. To block RBC invasion and achieve blood stage growth inhibition, antigens involved in this process needs to be targeted [4]. Two of the erythrocytic stage surface proteins of *Plasmodium* spp. named merozoite surface protein-1_19_ and apical membrane antigens-1 are the most promising candidates for malaria vaccine development due to the protective immune response against these parasite within the human and mammalian host [5, 6]. Both are important for merozoite invasion in RBC, highly immunogenic, can induce antibody in humans and contribute towards protective immunity [7, 8].

AMA-1 and MSP-1_19_ are well-characterized malaria vaccine candidates in *Plasmodium falciparum* and *Plasmodium vivax* [9, 10]. The C terminal 19 kDa region of MSP-1 remains on the surface of merozoites and initially plays role during adhesion of merozoites to RBCs [11–14]. The AMA-1 is an integral membrane protein expressed by merozoites and sporozoites [15]. This surface protein becomes crucial at the time of erythrocyte invasion as it is involved in the reorientation of merozoites [16]. Furthermore, during invasion AMA-1 binds to rhoptry neck protein (RON2) and forms the junction complex [16]. Several studies have reported that antibody against these antigens can inhibit the erythrocyte invasion by merozoites and it is associated with a decreased risk of malaria [6, 12, 17]. Individuals living in malaria endemic regions develop an effective immune response against the parasite and are less susceptible to malaria infection [18]. Moreover, population living in such endemic areas have been shown to possess anti-AMA-1 and anti-MSP-1_19_ antibodies, which increases with age [19–21]. Many in vitro and animal model studies have also shown that such antibodies can reduce parasite multiplication and protect from lethal infection [22–25].

The unique geographic position and diverse climate of India make it perfect for malaria transmission and presents challenges towards malaria control and elimination. An understanding of the host immune response, acquisition and maintenance of the antimalarial antibody to *P. vivax* vaccine candidate antigens in people living in malaria endemic areas is crucial for improving prospects on successful malaria vaccine development [26, 27]. Here, the antibody responses to recombinant *P. vivax* apical membrane antigen 1 (PvAMA-1) and merozoite surface antigen-1_19_ (PvMSP-1_19_) were investigated in individuals living at three geographically diverse malaria endemic regions of India. The immune status of the residents living in diverse *P. vivax* transmission area and factors associated with it has not been reported from India. Results of this study would be a support to evaluate the malaria vaccine development and elimination programme in India.

## Methods

### Study sites

**Fig. 1:**
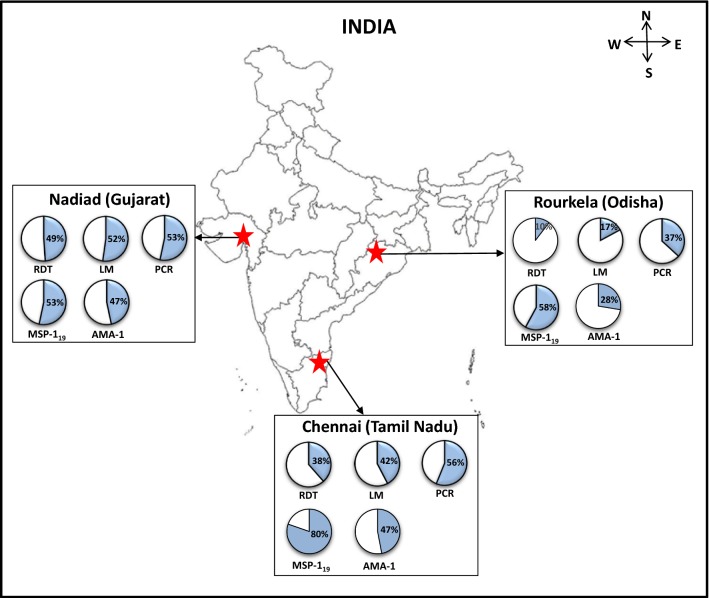
Map showing three study sites and the proportion of *Plasmodium vivax* infection evaluated by different methods. The pie graph represents the proportion (%) of *P. vivax* infection or seropositivity (%) for PvMSP-1_19_ and PvAMA-1 antigens. RDT is the rapid diagnostic test, LM is light microscopy and PCR is diagnostic PCR performed for species identification

The details of three field sites of the Center for the Study of Complex Malaria in India (CSCMi) i.e., Nadiad (Gujarat), Chennai (Tamil Nadu) and Rourkela (Odisha) have been described previously [28, 29]. These selected study sites represented different eco-epidemiological conditions, malaria vector system, transmission rates and relative prevalence of *P. vivax* and *P. falciparum*. Briefly, Chennai is the capital city of Tamil Nadu state (Fig. 1). Malaria transmission in Chennai is perennial due to humid and hot climate and malaria cases increases between July and October. *Anopheles stephensi* is the main malaria vector in Chennai and *P. vivax* is the dominant malaria species [30, 31]. In Chennai, annual parasite incidence (API, number of malaria cases per thousand population) was 2.34 in 2012 which reduced to 1.79 in 2013 [3]. Samples were collected from individuals enrolled at the Besant Nagar Malaria Clinic or in cross-sectional surveys conducted in few slums, urban dwellings and a large coastal community near the Besant Nagar area. Nadiad town is located in Kheda district of Gujarat state. Here, *P. vivax* and *P. falciparum* malaria occur throughout the year with a slightly higher prevalence of *P. vivax.* Nadiad has semi-arid and sub-tropical climate. In Nadiad, *Anopheles culicifacies* is the main malaria vector and API 2.5 observed in 2010 [3, 28] Samples were collected from individuals enrolled at a malaria clinic in Nadiad Civil Hospital and in cross-sectional survey conducted at nearby rural area of Nadiad town. Rourkela, is located in Sundargarh district of Odisha state and has a tropical wet and dry climate. *Anopheles culicifacies* and *Anopheles fluviatilis* are main malaria vectors with *P. falciparum* as a dominant malaria parasite species. It has highest API 7.57 among three selected sites in 2010 [3, 28]. Samples were collected from individuals enrolled at health clinic and from cross-sectional surveys conducted in rural areas of Rourkela.

### Sample collection and processing

Approximately 3 to 5 mL of blood was collected in EDTA vacutainers (Thermo Fisher, Massachusetts, USA) from each individual in cross-sectional surveys (CSS, N = 98) and clinic (CL, N = 136) at three field sites Chennai (CSS = 11, CL = 55), Nadiad (CSS = 32, CL = 60) and Rourkela (CSS = 55, CL = 21) during January 2013–May 2015. Plasma samples from 234 individuals (aged 1–70) were used for this study. Blood samples were centrifuged at 1500×*g* for 15 min at room temperature and plasma was removed and stored at − 80 °C. DNA extraction was performed using red blood cells by QiAamp DNA blood mini kits (Qiagen Inc., Valencia, CA). Individuals were enquired about their history of malaria in last 12 months, fever within last 2 weeks, do they had antimalarial drugs in last 2 weeks or taking any fever suppressants now, if they use bed nets, their age, gender were recorded.

Malaria infection was diagnosed by three test, rapid diagnostic test (RDT), blood smear microscopy, and polymerase chain reaction (PCR) for all samples collected in the cross sectional surveys and clinic studies. Malaria infection was determined by bivalent RDT (FalciVax, Zephyr Biomedicals, India) by finger prick blood at the time of enrollment, followed by microscopic examination of thick blood smear, stained with Giemsa by microscopy using a 100× oil immersion objective. Parasites were counted on the thick smear against 200–500 leucocytes (WBC) and expressed as parasites per microlitre of blood, using the WBC count if known, or assuming 8000 WBCs per microlitre of blood. A semi nested species specific PCR assay was used as described earlier [32] for molecular detection of *P. vivax* and/or *P. falciparum.* Infection of *Plasmodium* spp. was reconfirmed by diagnostic PCR at National Institute of Malaria Research Delhi and only *P. vivax* positive and malaria negative samples were selected for the study.

### Enzyme-linked immunosorbent assay (ELISA)

The total IgG antibody response against the *P. vivax* recombinant apical membrane antigen-1 (PvAMA-1) and the 19 kDa fragment of recombinant merozoite surface protein (PvMSP-1_19_) synthesized at London School of Hygiene and Tropical Medicine, London, UK as described previously [16, 33–35] were detected by an indirect ELISA as described previously [33, 36]. Briefly, recombinant PvAMA-1 (SalI strain) and PvMSP-1_19_ (Belem strain) antigens were diluted in coating buffer (0.15 M sodium carbonate, 0.034 M sodium bicarbonate, pH 9.6) to 0.5 µg/mL concentration and 50 µL of each diluted antigen were coated on 96 well ELISA plate (Immulon 4 HBX, Thermo Scientific). Plates were incubated overnight at 4 °C. After incubation, ELISA plates were washed with washing buffer (1× PBS, 0.05% Tween 20) (PBS/T) and blocked with 1% skimmed milk solution for 3 h. After washing plates with PBS/T solution, serum samples were added in duplicates at a final dilution of 1:1000 for PvMSP-1_19_ and 1:2000 for PvAMA-1 to each plate together with serial dilution of positive control (pooled hyper immune serum of 20 adults from Sepik, Papua New Guinea) and negative control (pooled serum samples from healthy individuals from USA who never experienced malaria). These plates were incubated overnight at 4 °C and washed with PBS/T solution. 50 µL of horseradish peroxidase-conjugated rabbit anti-human IgG (DAKO), diluted 1/5000 in PBS/T, were added in each wells, incubated at room temperature for 3 h and washed again with PBS/T solution. Ortho-phenylenediamine substrate (Sigma) was added 100 µL in each well and left in dark for 10–15 min at room temperature. 25 µL of stop solution (2 M H_2_SO_4_) was added in each well to stop the reaction and optical density (OD) at 492 nm was recorded using ELISA reader (NanoQuant, TECAN).

### Statistical analysis

Optical density (OD) values recorded in duplicate were averaged and normalized against blank wells values to adjust the background reactivity. To calculate seropositivity a separate cut-off was generated for each antigen. A plasma sample was considered seropositive when absorbance was higher than the mean OD plus twice of standard deviations (SD) of negative control (pooled serum samples from unexposed individuals in the USA). Titre values were calculated by plotting a titration curve, using the normalized OD values of serially diluted positive control in Microsoft-Excel as described previously [36]. All descriptive data (categorical variables) were expressed in number (N) and percentage (%). Seroprevalence was calculated as a percentage for participants who was seropositive either for PvAMA-1 or PvMSP-1_19_ separately and expressed as a proportion. 95% confidence interval (CI) was estimated using binomial distribution. All continuous normally distributed variables were expressed as mean ± SD while non-normally distributed variable was expressed as Median (P25 to P75). Normality assumption was checked using QQ plot, histogram and Kolmogorov–Smirnov test. Association between categorical variables was assessed using Chi-Square/Fisher exact test. In order to know the factors which may affect the seropositivity rate of PvAMA-1 and PvMSP-1_19_, association of various factors with seropositivity to PvAMA-1 and PvMSP-1_19_ was assessed using logistic regression, separately for each of the antigens. Followed by bi-variable analysis, a multivariable analysis was done using step-wise multivariable logistic regression. P-value of less than 0.05 was considered as significant and all statistical analysis was done using statistical software R 3.4 and Stata 15.0.

## Results

### Baseline characteristics

A total of 234 individuals were recruited from the three geographically diverse malaria endemic regions of India namely Chennai (n = 66), Nadiad (n = 92) and Rourkela (n = 76) in January 2013–May 2015. Age of the study participants ranged between 25 and 48 years (median = 32 years) in Chennai, 15.5–41 years (median = 29 years) in Nadiad and 11–35 years (median = 23 years) in Rourkela. The majority of participants were adults at each site. More than 60% study participants were male (n = 146). Overall, the bed net users were 70.51%. The bed net users were higher in Chennai (96.97%) and Nadiad (90.22%) while in Rourkela it was only 23.68%. Total 25% participants were having fever within last 2 weeks and were taking fever suppressant at the time of enrollment. Only 3.4% participants had taken anti-malarial drugs in last 2 weeks. In Rourkela, 25% of the study population recalled (by memory) that they may had one or more than one attack of malaria infection (either *P. vivax* or *P. falciparum*) in past 12 months, while in Chennai and Nadiad the percentage of a previous history of malaria was 25.76% and 5.43% respectively. Previous malaria exposure was higher and comparable in Rourkela and Chennai population as compare to Nadiad. More details of the study participants at each site are shown in Table 1.

**Table 1:** Baseline information

Characteristic	Study site			Overall (N = 234)
	Chennai (N = 66)	Nadiad (N = 92)	Rourkela (N = 76)	
Age (years)	32 (25–48)	29 (15.5–41)	23 (11–35)	29 (17–40)
Gender, male	41 (62.1)	60 (65.2)	45 (59.2)	146 (62.4)
Bed net use	64 (97)	83 (90.2)	18 (23.7)	165 (70.5)
Fever within last 2 weeks	20 (32.8)	29 (31.5)	10 (13.2)	59 (25.8)
Fever suppressant	54 (81.8)	29 (31.5)	19 (25.0)	102 (43.6)
Taken anti-malarial drugs in last 2 weeks	1 (1.6)	4 (4.3)	3 (3.9)	8 (3.4)
Malaria in last 12 months	17 (25.8)	5 (5.4)	19 (25.0)	41 (17.5)
RDT positive	25 (37.9)	45 (48.9)	8 (10.5)	78 (33.3)
Microscopy positive	28 (42.4)	48 (52.2)	13 (17.1)	89 (38.0)
PCR positive	37 (56.1)	49 (53.3)	28 (36.8)	114 (48.7)
Malaria cases				
Asymptomatic	28 (75.7)	27 (55.1)	25 (89.3)	80 (70.2)
Symptomatic	9 (24.3)	22 (44.9)	3 (10.7)	34 (29.8)
Parasite count				
Low (≤ 5000)	25 (86.2)	35 (72.9)	10 (58.8)	70 (74.5)
High (> 5000)	4 (13.8)	13 (27.1)	7 (41.2)	24 (25.5)

Number are expressed in n (%) and median (P25 to P75)

Overall *P. vivax* prevalence was 33.3%, 38.0% and 48.7% detected by RDT, microscopy and PCR methods, respectively. A total of 114 individuals were *P. vivax* malaria positive and 120 individuals were malaria negative as diagnosed by PCR. Malaria positivity as detected by PCR varied among settings, i.e., 37 (56.06%) in Chennai, 49 (53.26%) in Nadiad and 28 (36.84%) in Rourkela (Fig. 1). Irrespective of diagnostic method, higher *P. vivax* malaria prevalence was observed in Chennai and Nadiad than Rourkela.

Among the studied population, 70 (74.5%) subjects were asymptomatic (body temperature < 37.5 °C and PCR positive) and 24 (25.5%) subjects were symptomatic (body temperature > 37.5 °C and PCR positive). The number of asymptomatic subjects was higher at each site as compared to the number of symptomatic subjects. Overall 74.5% population were having low parasitaemia (≤ 5000, asexual parasites) and only 25.5% population were having high parasitaemia (> 5000, asexual parasites) (Table 1).

### Natural acquired antibody response against malaria antigens at three study sites in India

#### PvAMA-1 antibodies

**Fig. 2:**
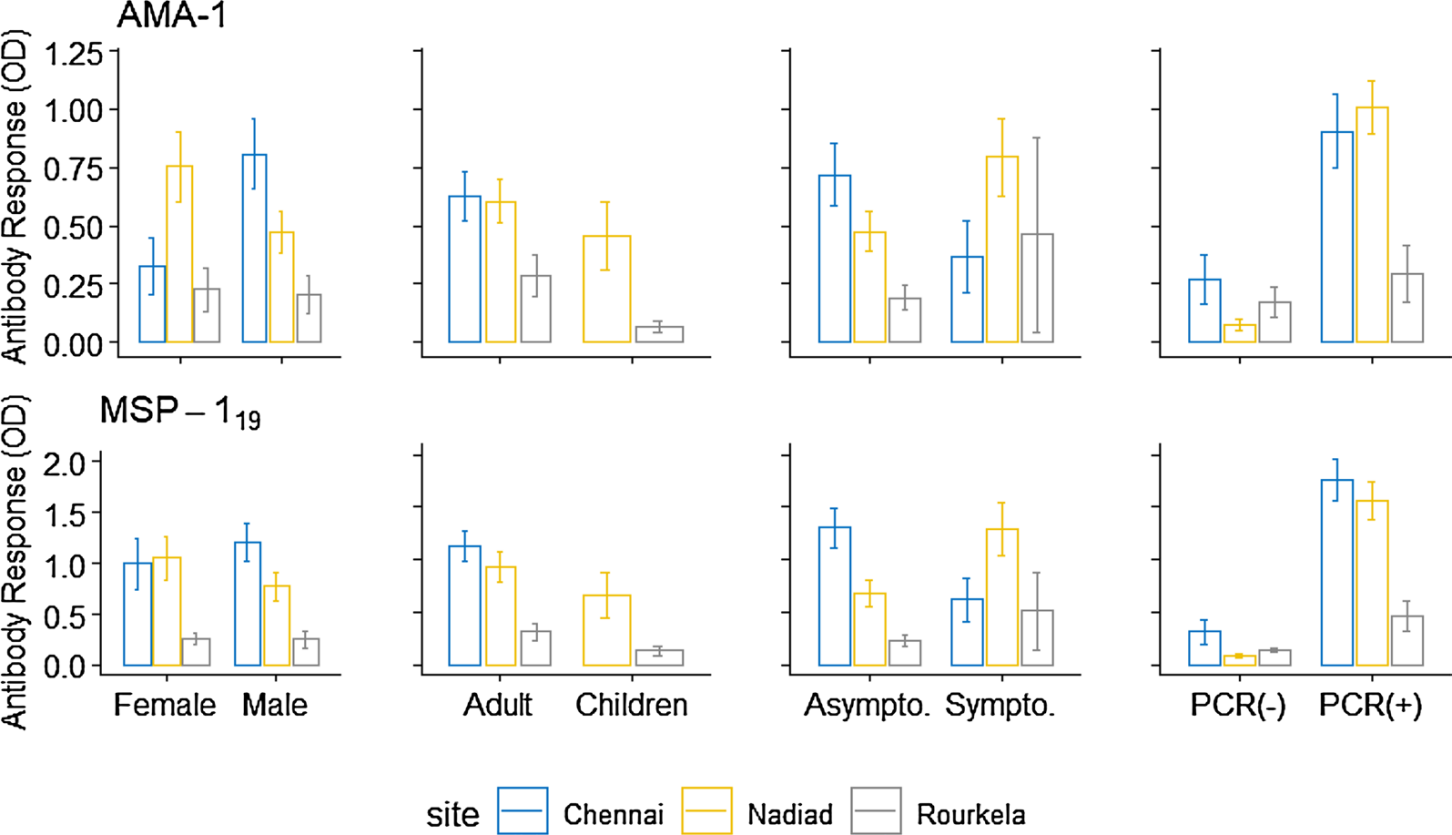
Antibody response against PvAMA-1 and PvMSP-1_19_ antigens as determined by optical density (OD) by ELISA, shown in different categories of gender, age, symptoms, and malaria -positivity by each sites using error bar plot. Here bar represents mean and error bar over it represents standard error

**Fig. 3:**
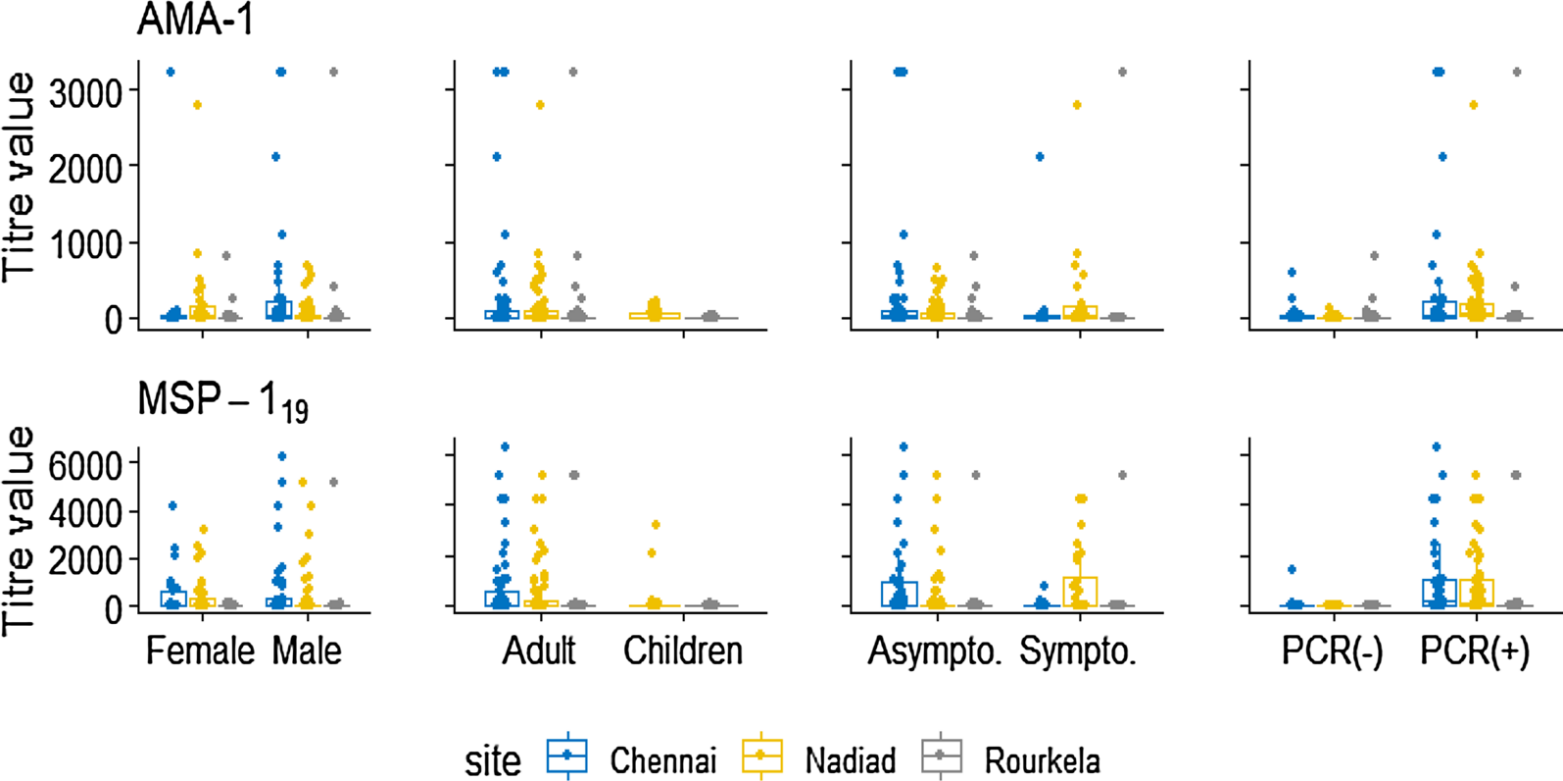
Antibody level against PvAMA-1 and PvMSP-1_19_ antigens as determined by titer value (IQR, interquartile range 25th–75th percentile) presented specific to gender, age-categories, symptoms and malaria positivity by each sites using Box-Whiskers plot

The antibody response whether evaluated as OD level, antibody titre or seroprevalence increased with increasing *P. vivax* prevalence. The overall PvAMA-1 seroprevalence was 40.6% (95% CI 34.4–47.1), which was higher in Chennai (47%, 95% CI 35.0–59.3) and Nadiad (46.7% 95% CI 36.6–57.1) than Rourkela (27.6%, 95% CI 18.6–39). Seroprevalence between male and female was varying across three sites, higher seroprevalence was observed for males (56.1%) than females (32.0%) in Chennai. While in other two sites, higher seroprevalence was observed for females (Nadiad 56.2%, Rourkela 35.5%) than males (Nadiad 41.7%, Rourkela 22.2%). For Nadiad and Rourkela, higher seroprevalence was observed in adults (≥ 15 years) (Nadiad 50%, Rourkela 35.3%) than children (< 15 years) (Nadiad 35%, Rourkela 12%) while in Chennai we had only adult participant whose seroprevalence was 47.0%. PvAMA-1 seroprevalence was higher in malaria positive (PCR positive) participants at each site as compared to malaria negative (PCR negative). Seroprevalence was higher in symptomatic individuals in Nadiad (81.8%), and Rourkela (66.7%) whereas in Chennai seroprevalence was higher in asymptomatic individuals (64.3%) as compared to symptomatic (55.6%). Sero-response was comparable between those who have high parasitaemia and those have low parasitaemia, and pattern was similar across sites (Table 2). Antibody titre for PvAMA-1 was higher in Chennai (mean = 248.34), followed by Nadiad (mean = 111.74) and Rourkela (mean = 67.11). Antibody response was higher in Chennai and Nadiad population as compared to Rourkela (Figs. 2 and 3).

**Table 2:** Antibody response against PvAMA-1 at three sites in India

Seroprevalence	Chennai		Nadiad		Rourkela		Total	
	n/N (%)	95% CI	n/N (%)	95% CI	n/N (%)	95% CI	n/N (%)	95% CI
Overall	31/66 (47.0)	35.0–59.3	43/92 (46.7)	36.6–57.1	21/76 (27.6)	18.6 –39.0	95/234 (40.6)	34.4–47.1
Gender								
Male	23/41 (56.1)	40.2–70.9	25/60 (41.7)	29.6–54.8	10/45 (22.2)	12.1–37.2	58/146 (39.7)	32.0–48.0
Female	8/25 (32.0)	16.0–53.7	18/32 (56.2)	38.1–72.9	11/31 (35.5)	20.1–54.5	37/88 (42.0)	32.0–52.7
Age (years)								
< 15	0	0	7/20 (35.0)	16.4–59.6	3/25 (12.0)	03.6–33.3	10/45 (22.2)	12.1–37.2
≥ 15	31/66 (47.0)	35.0–59.3	36/72 (50.0)	38.4–61.6	18/51 (35.3)	23.1 –49.7	85/189 (45.0)	38.0–52.2
PCR								
Positive	23/37 (62.2)	45.0 –76.7	39/49 (79.6)	65.5–88.9	13/28 (46.4)	28.2–65.7	75/114 (65.8)	56.5–74.0
Negative	8/29 (27.6)	13.1–47.5	4/43 (9.3)	03.4–23.0	8/48 (16.7)	08.3–30.5	20/120 (16.7)	10.9–24.5
Malaria								
Asymptomatic	18/28 (64.3)	44.1–80.4	21/27 (77.8)	57.0–90.2	11/25 (44.0)	25.2–64.7	50/80 (62.5)	51.2–72.6
Symptomatic	5/9 (55.5)	19.5–86.6	18/22 (81.8)	58.1–93.6	2/3 (66.7)	0.3–99.9	25/34 (73.5)	55.4–86.1
Parasitaemia								
Low	19/25 (76.0)	54.1–89.5	28/35 (80.0)	62.6–90.5	5/10 (50.0)	18.1–81.9	52/70 (74.3)	62.5–83.3
High	3/4 (75.0)	4.1–99.5	11/13 (84.6)	49.0–96.9	4/7 (57.1)	15.0–90.9	18/24 (75.0)	52.5–89.0

*CI* confidence interval

#### PvMSP-1_19_ antibodies

The overall seroprevalence for PvMSP-1_19_ was 62.4% (95% CI 56–68.4) which was higher than PvAMA-1 seroprevalence (40.6%, 95% CI 34.4–47.1) across three study sites. PvMSP-1_19_ seroprevalence was higher in Chennai (80.3%), compared to Nadiad (53.3%) and Rourkela (57.9%) (Table 3). Higher seroprevalence was observed in females in Nadiad (62.5%) and Rourkela (61.3%) while in Chennai higher seroprevalence was observed in males (82.9%) as compared to females (76.0%). Between two age groups higher seroprevalence was found in adults at all three study sites (Fig. 2). Seroprevalence to PvMSP-1_19_ antigen in *P. vivax* infected (PCR confirmed) individuals was higher in Chennai (83.8%) and Nadiad (85.7%), but Rourkela showed slightly high seropositivity in malaria negative individuals (60.2%) as compared to *P. vivax* positive individuals (53.6%). Seroprevalence was higher in asymptomatic individuals as compared to symptomatic patients in Nadiad (92.6%) and Chennai (85.7%), whereas in Rourkela seroprevalence was higher in symptomatic individuals (100%). Antibody response between high parasitaemia and low parasitaemia group was almost equal at each site (Table 3). Antibody titre for PvMSP-1_19_ antigen was higher in Chennai (mean = 593.70) and Nadiad (mean = 461.46) than Rourkela (mean = 143.36). Overall antibody titre was higher for PvMSP-1_19_ (mean = 395.44) as compared to PvAMA-1 (mean = 135.77).

**Table 3:** Antibody response against PvMSP-1_19_ at three sites in India

Seroprevalence	Chennai		Nadiad		Rourkela		Total	
	n/N (%)	95% CI	n/N (%)	95% CI	n/N (%)	95% CI	n/N (%)	95% CI
Overall	53/66 (80.3)	68.6–88.4	49/92 (53.3)	42.9–63.4	44/76 (57.9)	46.3–68.7	146/234 (62.4)	56.0–68.4
Gender								
Male	34/41 (82.9)	67.5–91.9	29/60 (48.3)	35.7–61.2	25/45 (55.5)	40.4–69.7	88/146 (60.3)	52.0–68.0
Female	19/25 (76.0)	54.1–89.5	20/32 (62.5)	43.9–78.0	19/31 (61.3)	42.4–77.3	58/88 (65.9)	55.2–75.2
Age (years)								
< 15	0	–	10/20 (50.0)	27.7–72.3	11/25 (44.0)	25.2–64.7	21/45 (46.7)	32.2–61.7
≥ 15	53/66 (80.3)	68.6–88.4	39/72 (54.2)	42.4–65.5	33/51 (64.7)	50.3–76.9	125/189 (66.1)	59.0–72.6
PCR125/189 (66.1)								
Positive	31/37 (83.8)	67.4–92.8	42/49 (85.7)	72.4–93.2	15/28 (53.6)	34.3–71.8	88/114 (77.2)	68.5–84.1
Negative	22/29 (75.9)	56.0–88.6	7/43 (16.3)	07.7–31.1	29/48 (60.4)	45.6–73.5	58/120 (48.3)	39.4–57.4
Malaria								
Asymptomatic	24/28 (85.7)	66.0–94.9	25/27 (92.6)	72.8–98.3	12/25 (48.0)	28.4–68.2	61/80 (76.2)	65.5–84.4
Symptomatic	7/9 (77.8)	33.0–96.1	17/22 (77.3)	53.5–90.9	3/3 (100)	–	27/34 (79.4)	61.6–90.2
Parasitaemia								
Low	23/25 (92)	70.9–98.2	30/35 (85.7)	68.9–94.2	5/10 (50.0)	18.1–81.9	58/70 (82.8)	71.9–90.1
High	4/4 (100)	–	12/13 (92.3)	53.1–99.2	3/7 (42.8)	9.1–84.9	19/24 (79.2)	56.8–91.7

*CI* confidence interval

### Factors associated with seropositivity to PvAMA-1 and PvMSP-1_19_

A total of ten factors (sex, age, malaria positivity by PCR, symptomatic and asymptomatic malaria, Parasitaemia, fever within 2 weeks, taking fever suppressants, anti-malarial drugs, malaria in last 12 months and diverse eco-epidemiological study sites) were considered as potential predictors of seropositivity for both antigens. Among these, four predictors (age, malaria positivity using PCR, fever in last 2 weeks, and study sites) were found statistical significant in bi-variate analysis for PvAMA-1. In multivariable analysis, done by step-wise logistic regression, two predictors viz. age and malaria positivity by PCR were found to significantly associated with seropositivity of PvAMA-1. Odds of being seropositive to PvAMA-1 for adult (≥ 15 years) was almost four fold [OR (95% CI) 4.12 (1.76–10.11)] higher than children (age < 15 years). Odds of being seropositive to PvAMA-1 for malaria diagnosed person using PCR was almost 13 [OR (95% CI) 13.02 (6.52–22.50)] times more than the person who do not have malaria (Table 4).

**Table 4:** Factors associated with seropositivity to PvAMA-1

Factors	Seropositive, n/N (%)	Unadjusted		Adjusted	
		OR (95% CI)	P value	OR (95% CI)	P value
Gender					
Male	58/146 (39.73)	Ref.	0.726	^a^	
Female	37/88 (42.05)	0.91 (0.53–1.55)			
Age					
< 15	10/45 (22.22)	Ref.	0.007	Ref.	0.001
≥ 15	85/189 (44.97)	2.86 (1.34–6.11)		4.12 (1.76–10.11)	
PCR					
Positive	75/114 (65.79)	Ref.	< 0.001	Ref.	< 0.001
Negative	20/120 (16.67)	9.61 (5.19–17.81)		13.02 (6.52–25.50)	
Malaria cases					
Asymptomatic	69/80 (72.63)	Ref.	0.119	^a^	
Symptomatic	26/34 (27.37)	1.64 (0.88–3.04)			
Parasite count					
Low	52/70 (74.29)	Ref.	0.945	^a^	
High	18/24 (75.00)	1.04 (0.36–3.02)			
Fever within last 2 week					
No	55/170 (32.35)	Ref.	< 0.001	^a^	
Yes	38/59 (64.41)	3.78 (2.03–7.05)			
Fever suppressant					
No	47/132 (35.61)	Ref.	0.078	^a^	
Yes	48/102 (47.06)	1.61 (0.95–2.72)			
Taken anti-malarial drug					
No	90/224 (40.18)	Ref.	0.580	^a^	
Yes	4/8 (50.00)	1.48 (0.36–6.11)			
Malaria in last 12 months					
No	74/193 (38.34)	Ref.	0.130	^a^	
Yes	21/41 (51.22)	1.69 (0.85–3.32)			
Study sites					
Rourkela	21/76 (27.63)	Ref.		^a^	
Chennai	31/66 (46.97)	2.31 (1.15–4.65)	0.018		
Nadiad	43/92 (46.74)	2.30 (1.20–4.39)	0.012		

*OR* odds ratio

^a^Omitted in multivariable analysis

Similar analysis was done for second antigen i.e. PvMSP-1_19_. In this case, six factors (age, malaria positive by PCR, fever within 2 weeks, fever suppressant, malaria in last 12 month and study sites) out of ten factors showed association with seropositive to PvMSP-1_19_ in bi-variable analysis. In multivariable analysis four predictors (viz. age, malaria positive by PCR, fever within last 2 weeks, and malaria in last 12 months) were identified significantly associated with seropositivity of PvMSP-1_19_. Odds of being seropositive to PvMSP-1_19_ for adult (≥ 15 years) was almost two fold [OR (95% CI) 2.70 (1.25–5.85)] higher than children (age < 15 years). Odds of being seropositive to PvMSP-1_19_ for malaria diagnosed person using PCR was also almost two [OR (95% CI) 2.44 (1.29–4.63)] times more than the person who do not have malaria. Odds of being seropositive to PvMSP-1_19_ of those who experienced fever in last 2 weeks five times [OR (95% CI) 5.70 (2.19–14.84)] more than those who were free from fever in last 2 weeks. Odds of being seropositive to PvMSP-1_19_ of those who experienced malaria fever in last 12 months was three times [OR (95% CI) 3.65 (1.48–9.03)] higher than those who did not experience malaria in last 12 months (Table 5).

**Table 5:** Factors associated with seropositivity to PvMSP-1_19_

Factors	Seropositive, n/N (%)	Unadjusted		Adjusted	
		OR (95% CI)	P value	OR (95% CI)	P value
Gender					
Male	88/146 (60.27)	Ref.	0.389	^a^	
Female	58/88 (65.91)	0.78 (0.45–1.36)			
Age					
< 15	21/45 (46.67)	Ref.	0.017	Ref.	0.011
≥ 15	125/189 (66.14)	2.23 (1.15–4.31)		2.70 (1.25–5.85)	
PCR					
Positive	88/114 (60.27)	Ref.	0.000	Ref.	0.006
Negative	58/120 (39.73)	3.62 (2.05–6.37)		2.44 (1.29–4.63)	
Malaria cases					
Asymptomatic	61/80 (76.2)	Ref.	0.713	^a^	–
Symptomatic	27/34 (79.4)	1.20 (0.45–3.19)			
Parasite count					
Low	58/70 (82.86)	Ref.	0.686		
High	19/24 (79.17)	0.79 (0.24–2.52)			
Fever within last 2 week					
No	89/170 (52.35)	Ref.	0.000	Ref.	< 0.001
Yes	53/59 (89.83)	8.04 (3.28–19.70)		5.70 (2.19–14.84)	
Fever suppressant					
No	71/132 (53.79)	Ref.	0.002	^a^	–
Yes	75/102 (73.53)	2.39 (1.37–4.17)			
Taken anti-malarial drug					
No	136/224 (60.71)	NA	–	^a^	–
Yes	8/8 (100)				
Malaria in last 12 months					
No	113/193 (58.55)	Ref.	0.011	Ref.	0.005
Yes	33/41 (80.49)	2.92 (1.28–6.65)		3.65 (1.48–9.03)	
Study sites					
Rourkela	44/76 (57.89)	Ref.		^a^	–
Chennai	53/66 (80.30)	2.96 (1.39–6.33)	0.018		
Nadiad	49/92 (53.26)	0.83 (0.45–1.53)	0.012		

*OR* odds ratio

^a^Omitted in multivariable analysis

## Discussion

In India, insight to naturally acquired antibody response to leading *P. vivax* vaccine candidate antigens for development of an effective vaccine that can work in diverse regions is very limited. The present study is first attempt to investigate antibody response against *P. vivax* vaccine candidate antigens PvAMA-1 and PvMSP-1_19_ in individuals living in three diverse eco-epidemiological regions of India, with differing prevalence of *P. vivax* and *P. falciparum*. Total IgG antibody response was determined against these two antigens in 234 individuals living in Chennai, Nadiad and Rourkela. In these diverse eco-epidemiological study site, heterogeneity between the RDT (33.3%), microscopy (38.0%) and PCR (48.7%) was observed in screening of *P. vivax* parasite prevalence. Lower parasite prevalence by RDT was observed at all three sites as compared to microscopy and PCR. Higher seroprevalence or antibody titre against these two antigens was observed at Chennai and Nadiad where *P. vivax* prevalence is high as compared to Rourkela (*P. falciparum* dominant area). Heterogeneity in seropositivity against these two antigens was observed at all three study sites and the overall seroprevalence for PvMSP-1_19_ was higher (62.4%) as compared to PvAMA-1 (40.6%). A total of 35.9% individuals were seropositive for both the antigens and seropositivity was higher in Chennai (45.4%) and Nadiad (41.3%) as compared to Rourkela (21%). Whereas, 32.9% individuals were not showing any antibody response against PvAMA-1 and PvMSP-1_19_ antigens, might have slower immune response which stimulated antibodies but not up to the detectable level. This study observed PvMSP-1_19_ to be highly immunogenic when compared to PvAMA-1 at all three study sites and similar observation was reported in previous immune-epidemiologic study conducted in north India [26] and other countries like Brazil and Haiti [11, 31, 37–39]. A genome-scale protein microarray study of seroreactivity to different *P. vivax* and *P. falciparum* antigens conducted at these three field sites and in Goa (Southwest India) also reported merozoites surface proteins as the most immunogenic antigen in *P. vivax* and reported apical membrane antigen as most immunogenic in *P. falciparum* [40, 41]. One of the possible reason for higher immunogenicity for PvMSP-1_19_ could be its conserved genetic nature, which were reported in many studies [21, 42–47] and the similar observation were recorded in our field isolates collected from Chennai, Nadiad and Rourkela (unpublished data). In contrast, *Pvama*-*1* gene found to be highly polymorphic at these three study sites (unpublished data) and also in various previous studies [48–54]. Another possible reason could be higher exposure of PvMSP-1_19_ on the surface of infected RBCs until the end of the intracellular cycle [27]. Antibody response was higher in *P. vivax* infected individuals against the PvAMA-1 antigen at all three study population. Interestingly, in Rourkela, seropositivity to PvMSP-1_19_ antigen was slightly higher in *P. vivax* uninfected individuals (60.4%, PCR negative) as compared to infected individuals (53.6%, PCR positive), suggest antibody response against the PvMSP-1_19_ antigen once acquired due to cumulative exposure of the parasite over time persist for so many years [55]. In Rourkela 25% of participants had previous exposure (in 12 months) to *P. vivax* or *P. falciparum* malaria indicates earlier exposure of malaria parasite can enhance the immune response. Another possibility is mixed infections and cross-reactivity between antibodies formed towards *P. falciparum* MSP-1_19_ [56, 57]. Chances of being seropositive to *P. vivax* MSP-1 were highly associated with being seropositive to *P. falciparum* MSP-1, described by a previous study [56] and this could be the reason of higher seropositivity against PvMSP-1_19_ antigen in Rourkela where *P. falciparum* in dominant species. However, cross reactivity between antibodies formed against recombinant PvAMA-1 and PfAMA-1 antigen is very limited [58] and that could be the reason of less seropositivity in PvAMA-1 antigens as compared to PvMSP-119 in Rourkela. A protein array-based immune surveillance study conducted in Goa, in Southwest India where *P. vivax* is dominant species reported strong IgG response towards *P. falciparum* antigens [41]. These results highlight the need to better understand antigenic cross reactivity in areas where both *P. falciparum* and *P. vivax* co-exist. In Chennai and Nadiad population antibody response against the PvMSP-1_19_ antigen was higher in *P. vivax* infected individuals. Prevalence of *P. vivax* relapse infections in an area may provide protective immunity [59], however the study did not observe any relapse cases.

Antibody response was compared in children and adults from two sites Nadiad and Rourkela as Chennai had an insufficient number of children enrolled. The antibody response against PvAMA-1 and PvMSP-1_19_ antigen was significantly higher in adults as compared to children at each study site which clearly indicate age acquired immunity. The immune response increases with age due to repeated exposure of malaria parasite [60, 61]. In addition, it was noted that the antibody response against the two antigens was higher in females as compared to males in Nadiad and Rourkela though these differences were not statistically significant. The possible reason for the differences could be the females were more often infected, therefore they might show high antibody response against the antigens. This pattern was not observed in Chennai, where antibody response was higher in males as compared to females, probably due to life style and socio-economic factor when compared to Nadiad and Rourkela. Results indicate a higher antibody response in asymptomatic individuals as compared to symptomatic, though these differences were not statistically significant. Individuals living in malaria-endemic areas are able to control parasitaemia through the immune mechanism and could remain asymptomatic [62, 63], which could be the reason for increased asymptomatic malaria cases in the study population. Antibody response against PvMSP-1_19_ and PvAMA-1 antigens in individuals having low parasitaemia was higher (insignificant) as compared to individuals having high parasitaemia in all three study sites. This may indicate that parasitaemia cannot be correlated with antibody response and antibodies against blood-stage antigens are developed in most individuals living in malaria endemic areas [63].

Total ten factors which could be associated with seropositivity namely sex, age, malaria positivity, symptomatic and asymptomatic malaria cases, parasitaemia, fever, fever suppressants, anti-malarial drugs, past malaria infection and heterogeneous malaria transmission sites were evaluated. Four factors age, malaria positivity (by PCR), fever (within last 2 week) and past malaria infection (in last 12 months) were significantly associated with seropositivity to PvMSP-1_19_. Whereas, only two factors age and malaria positivity (by PCR) were significantly associated with seropositivity to PvAMA-1. Seropositivity to PvMSP-1_19_ and PvAMA-1 was higher in individuals who were having fever within last 2 weeks in Chennai, Nadiad and Rourkela and were malaria positive (by PCR), which clearly indicates that individuals with malaria infection naturally produce antibodies against these two antigens at each site resulted in higher antibody response. Individuals with past history of malaria in last 12 months recalled by memory were significantly associated with seropositivity to PvMSP-1_19_ as compared to individuals who were not infected by malaria in last 12 months at each site, indicates that frequent infection increase the antibody response and boosted with subsequent attack of malaria infection [18]. Fever within last 2 weeks and diverse study sites were significantly associated with seropositivity to PvAMA-1 and PvMSP-1_19_ antigens in logistic regression analysis but omitted in multivariable analysis and larger sample size may be required to observe the association. Other factors such as symptomatic and asymptomatic malaria cases, parasitaemia, fever suppressants, and anti-malarial drugs were not significantly associated with seropositivity for both the antigens at three sites. This study has few limitations; low number of study participants and the study has lower number of children at each study site as compared to adults.

Limited studies were conducted in the Asia–Pacific region to identify immune response in a geographical diverse population with different *P. vivax* endemicity [64–68]. Basic understanding of antibody response against the vaccine antigens in different geographical areas is important to assess the effectiveness of malaria vaccine. This study clearly demonstrated that individuals living in three malaria endemic areas of India greatly vary in their antibody response to these two leading blood-stage vaccine antigens. PvMSP-1_19_ is highly immunogenic and recognized more strongly in different geographical population. The varied antibody response observed between two antigens in the present study could be human genetic background, antigenic polymorphism, structural differences in the antigens, differential responsiveness and differences in *P. vivax* transmission in the study sites, which needs further investigation.

## Conclusion

In conclusion, it is evident that *P. vivax* MSP-1_19_ is highly immunogenic during natural infection in individuals living in three geographically diverse malaria endemic regions of India. There is heterogeneity in antibody response to PvMSP-1_19_ and PvAMA-1 antigen among three populations. Results obtained here have implications for understanding human immunity to malaria antigens in different populations and could be helpful in malaria elimination programme and vaccine development.

## Data Availability

All data generated or analysed during this study are included in this published article.

## Abbreviations

MSP-1_19_: merozoite surface protein-1_19_
PvAMA-1: apical membrane antigen-1
LM: light microscopy
PCR: polymerase chain reaction
ELISA: enzyme linked immunosorbent assay
OD: optical density
OR: odds ratio
CI: confidence interval
